# Evidence of horizontal transfer of non-autonomous *Lep*1 *Helitrons* facilitated by host-parasite interactions

**DOI:** 10.1038/srep05119

**Published:** 2014-05-30

**Authors:** Xuezhu Guo, Jingkun Gao, Fei Li, Jianjun Wang

**Affiliations:** 1 College of Horticulture and Plant Protection, Yangzhou University, Yangzhou, 225009, China; 2 College of Plant Protection, Nanjing Agricultural University, Nanjing, 210095, China

## Abstract

Horizontal transfer (HT) of transposable elements has been recognized to be a major force driving genomic variation and biological innovation of eukaryotic organisms. However, the mechanisms of HT in eukaryotes remain poorly appreciated. The non-autonomous *Helitron* family, *Lep*1, has been found to be widespread in lepidopteran species, and showed little interspecific sequence similarity of acquired sequences at 3′ end, which makes *Lep*1 a good candidate for the study of HT. In this study, we describe the *Lep*1-like elements in multiple non-lepidopteran species, including two aphids, *Acyrthosiphon pisum* and *Aphis gossypii*, two parasitoid wasps, *Cotesia vestalis*, and *Copidosoma floridanum*, one beetle, *Anoplophora glabripennis*, as well as two bracoviruses in parasitoid wasps, and one intracellular microsporidia parasite, *Nosema bombycis*. The patchy distribution and high sequence similarity of *Lep*1-like elements among distantly related lineages as well as incongruence of *Lep*1-like elements and host phylogeny suggest the occurrence of HT. Remarkably, the acquired sequences of both *NbLep*1 from *N. bombycis* and *CfLep*1 from *C. floridanum* showed over 90% identity with their lepidopteran host *Lep*1. Thus, our study provides evidence of HT facilitated by host-parasite interactions. Furthermore, in the context of these data, we discuss the putative directions and vectors of HT of *Lep*1 *Helitrons*.

Transposable elements (TEs) are prevalent in the genomes of almost all eukaryotes and are traditionally categorized based on their mode of transposition as class-I elements or retrotransposons and class-II elements or DNA transposons^1^. Copy and paste retrotransposons replicate via an RNA intermediate, which is reverse transcribed prior to its reintegration into the genome, whereas DNA transposons move through a single or double-stranded DNA intermediate and were divided into three major subclasses including the classic “cut-and-paste” transposons, rolling-circle (RC) transposons called *Helitrons*, and *Mavericks*, whose mechanism of transposition is not yet well characterized, but that likely replicate using a self-encoded DNA polymerase^2^.

The inherent mobility and replication abilities of TEs make them particularly prone to transfer horizontally between organisms to avoid co-evolved host suppression mechanisms leading to vertical inactivation^3^,^4^. Horizontal transfer (HT) can be defined as the exchange of genetic material between species by nonvertical inheritance without the aid of any form of sexual mechanism^5^. Over 200 solid cases of horizontal transfers of TEs (horizontal transposon transfer or HTT) have been described so far in multicellular eukaryotes^6^,^7^ with the majority of HTT cases involving drosophilid flies, and it is believed that TEs rely heavily on HT for their propagation and maintenance throughout evolution^8^,^9^. However, despite mounting examples of HTT, the unequivocal confirmation of any specific mechanism acting to shuttle DNA among eukaryotes remains poorly appreciated.

*Helitrons*, a new superfamily of transposons, have recently been uncovered by the computational analysis of genomic sequences of *Arabidopsis thaliana*, *Oryza sativa* and *Caenorhabditis elegans*^10^. Unlike traditional class DNA TEs, *Helitrons* are unique in that they do not produce target site duplications on their integration into the host genome and do not contain terminal repeats, and thus are difficult to be identified^11^,^12^,^13^. However, *Helitrons* have conserved sequence features including a “TC” motif on the 5′-end and a “CTRR” motif on the 3′-end, and contain a palindromic sequence of 16–20 bp near the 3′-terminus, which can form a hairpin structure^10^,^14^. In addition, *Helitrons* tend to insert preferentially between host nucleotides adenine and thymidine^10^,^15^. The non-autonomous *Helitrons*, *Lep*1, were originally identified within intron and untranslated regions from eight lepidopteran species^16^, and subsequently described as lepidopteran-specific common sequence 3(LSCS3)^17^. Recent study showed that *Lep*1 *Helitrons* were widespread in more than 30 lepidopteran species, and estimated to occupy 1.3 × 10^−5^ of the *Bombyx mori* genome sequence^18^.

Although an increasing number of *Lep*1 elements are being identified in lepidopteran genomes, little is known about *Lep*1 in non-lepidopteran insect species. In this study, we report the presence of *Lep*1-like elements in several non-lepidopteran insect species and other distantly related organisms. Our results suggested that the *Lep*1 *Helitrons* can undergo horizontal transfer by diverse means.

## Results and Discussion

### Evolutionary dynamics of *Lep*1 in *Helicoverpa armigera* and its related species

While *Lep*1 *Helitrons* have been previously described in multiple lepidopteran insects, the evolutionary dynamics of *Lep*1 had not been further investigated. In this study, a *Lep*1-like sequence (named *HaLep*1_1) was identified in *H. armigera* by genome walking and subsequent sequence analysis. The *HaLep*1_1 element is 193 bp in length and located at 756 bp upstream of the translation start codon of the *CYP6AE12* gene in the reverse orientation. A total of 21 full length sequences with high homology to *HaLep*1_1 were identified from non-redundant database, and named *HaLep*1_2- *HaLep*1_22 (Table S1). Figure S1 shows the alignment of these sequences. As shown in Figure S1, these sequences present the typical structural features of the *Lep*1 elements: almost all *HaLep*1 copies have characteristic 5′-TC and 3′-CTRY nucleotide termini as well as CTRR motif at the 3′ end of acquired sequence. The integration occurs precisely between the host A and T nucleotides, without duplications or deletions of the target sites, consistent with the RC mechanism. The phylogeny was constructed based on nucleotide sequences of all these *HaLep*1 elements. Neighbor-joining (NJ) analysis demonstrated the presence of three clear major lineages (Fig. S2), designated Lineage A (*HaLep*1A), Lineage B (*HaLep*1B), and Lineage C (*HaLep*1C), among which, 6 elements form lineage *HaLep*1A, while *HaLep*1B and *HaLep*1C were represented by 6 and 9 elements, respectively. Notably, *HaLep*1 elements from Lineage A and Lineage B showed relatively high identity with 134 bp *Lep*1 consensus sequence (83%–89%), while *HaLep*1 elements from Lineage C showed only 68% to 78% identity with *Lep*1 consensus sequence (Table S1). These results suggested that *HaLep*1 lineages might transfer independently into the genome of *H. armigera*.

The *HaLep*1_1 sequence was used as a query to search against nucleotide (*nr/nt*) and EST (*est_others*) collections to detect sequences with high identity with *HaLep*1_1 in lepidopteran species other than *H. armigera*. The result showed that *HaLep*1_1 sequence shared the highest similarity with two species of Heliothinae including *Helicoverpa zea* and *Heliothis virescens*. For example, three sequences from *H. zea* (accession number: EF152213, EF152207 and HQ840515) were identified from nucleotide (*nr/nt*) database to have over 93% identity with *HaLep*1_1. A total of 103 matches were detected in *H. virescens* EST database with an E-value less than 1e^−50^. Representative examples of these sequences are shown in Figure S3. Remarkably, the acquired sequence at 3′ end was only found in *H. zea* and *H. virescens*. Further analysis showed that the acquired sequences at 3′ end of all other *HaLep1* elements were also conserved only in *H. zea* and *H. virescens* (Table S2), suggesting that the acquired sequence was unique to *H. armigera* and its closely related species. These results consist with previous finding that the acquired sequence at 3′ end of *Lep*1 elements shared little interspecific sequence similarity, while high similarity was only found within species or closely related species^18^.

To understand whether *HaLep*1 elements mobilized recently, the insertion polymorphism was assessed experimentally or by homology searches. The results of PCR and subsequent sequencing of DNA products showed that in samples of 12 individuals, the percentage of individuals with the band for *HaLep*1_1 insertion was 25% (Fig. S4A). Paralogous or orthologous empty sites were also analyzed using homology searches. The results showed that no *Lep*1-like sequence was found in paralogous sites of *HaLep*1_20 (accession number: FP340435) in *H. armigera* as well as in orthologous site of *HaLep*1_8 in *H. zea* (accession number: DQ788839) (Fig. S4B, C). The *H. armigera* is a pest widespread across the Old World from the Western Pacific to the Canary Islands, while *H. zea* is found throughout the warm regions of the New World and in Hawaii^19^, and is recently thought to be derived from a founder population of *H. armigera* approximately 1.5 million years ago^20^. The intra-species insertion polymorphism of *HaLep*1_1 suggested a very recent transposition. The insertion polymorphism of *HaLep1_8* in two different but closely related species suggested that *HaLep1_8* might horizontally transfer into a common ancestor of *H. armigera* and *H. zea*, and the absence of orthologous copy in *H. zea* was due to the fact that the element had been actively transposing some time after the split of these two species, or to the differential fixation or loss of ancestrally polymorphic insertions in these two species. Further research is necessary to identify the parent TE of the non-autonomous *HaLep*1 elements.

### Identification of *Lep*1-like sequences in non-lepidopteran species

To characterize the distribution of *Lep*1-like elements in non-lepidopteran insect species, *Lep*1 consensus sequence was used as query in Blastn searches against insect genome assembly. While no significant hits were detected in the genomes of red flour beetle, *Tribolium castaneum* (Coleoptera: Tenebrionidae), the blood-sucking bug, *Rhodnius prolixus* (Hemiptera: reduviidae), the human body louse, *Pediculus humanus* (Phthiraptera: Pediculidae), the honey bee, *Apis mellifera* (Hymenoptera: Apidae), the parasitoid wasp *Nasonia vitripennis* (Hymenoptera: Pteromalidae), and six ants (Hymenoptera: Formicidae) including *Camponotus floridanus*, *Linepithema humile*, *Pogonomyrmex barbatus*, *Atta cephalotes*, *Harpegnathos saltator*, and *Solenopsis invicta*, our Blastn search detected 138 hits with ≥70% identity to the query over >100 bp in the pea aphid, *Acyrthosiphon pisum* (Hemiptera: Aphididae) genome assembly (AphidBase 2.1) (Table S3). However, because of the presence of many chimaeric elements, the acquired sequence regions as well as the proper boundaries of these *Lep1*-like sequences could not be precisely defined by multiple sequence alignment. Interestingly, one 662 bp EST sequence from the cotton aphid, *Aphis gossypii* (accession number: GW506388) also showed high identity with *Lep*1 consensus sequence (89%) as well as *HaLep*1_8 (90%).

*Lep*1 consensus sequence was further used as query in Blastn searches against all the species with sequences deposited in the GenBank databases. A total of 278 significantly similar sequences to *Lep1* (≥70% identity to the query over >100 bp) were identified in the genome shotgun sequence of *Anoplophora glabripennis* (Coleoptera: Cerambycidae). These sequences were subjected to pairwise alignment to reveal the boundaries and evaluated for the presence of structural features typical of *Lep*1 *Helitrons,* of these, a total of 175 full length elements were identified and named *AglaLep*1_1 to *AglaLep*1_175 (Table S4). The consensus sequence of the *AglaLep*1 is 209 bp long, shared 86% similarity with *Lep*1. It also has characteristic 5′-TC and 3′-CTRY nucleotide termini as well as CTRR motif at the 3′ end of 65 bp acquired sequence. Comparative analysis showed that the match between the *AglaLep*1 elements and their consensus sequence ranged from 95% to 100% (excluding indels), with a median similarity of 98%, suggesting a recent transposition activity.

Blastn searches using the *Lep1* consensus sequence as a query also yielded several significant hits in two parasitoid wasps, *Cotesia vestalis* and *Copidosoma floridanum*, as well as one microsporidia parasite, *Nosema bombycis* (Table 1). For example, two elements from *C. vestalis* (*CvLep*1_1 and *CvLep*1_2) showed 90% and 86% identity with *Lep*1, which are 190 bp and 201 bp in length including 62 bp and 65 bp acquired sequence, respectively. In *C. floridanum*, two full length copies of *Lep*1-like elements, *CfLep*1_1 and *CfLep*1_2, were identified, which are 253 bp and 236 bp in length including 122 bp and 100 bp acquired sequence, and showed 75% and 69% identity with *Lep*1, respectively. Three full length copies of *Lep*1-like elements were also found in *N. bombycis* (*NbLep*1_1- *NbLep*1_3), which are 445, 208, 218 bp in length including 314 bp, 76 bp and 84 bp acquired sequence, and showed 93%, 83%, 84% identity with *Lep*1, respectively.

It is also noteworthy that we identified highly similar sequences in two polydnaviruses (PDVs), which are symbiotically associated with hymenopteran wasps, including three copies from *C. vestalis* bracovirus (*CvBVLep*1_1-*CvBVLep*1_3), four copies from Kitale (CsKBVLep1_1-CsKBVLep1_4) and Mombasa (*CsMBVLep*1_1- *CsMBVLep*1_4) strains of *Cotesia sesamiae* bracovirus (Table 1). These elements vary in size from 196 bp (*CsMBVLep*1_1) to 344 bp (*CsKBVLep*1_4). Pairwise comparisons of individual elements reveal high sequence identity (82%–94%) with *Lep*1 consensus sequence (Table 1).

Overall, our BLAST searches detected significantly similar sequences to *Lep*1 element in other non-lepidopteran species. While cross-species contamination is a concern, our Blastx analysis of the flanking sequences of the representative non-lepidopteran *Lep*1 elements did not find any evidence of contamination (Table S5). The largest number of sequences with significant similarity to *Lep*1 was identified in *A. pisum* and *A. glabripennis*. However, this is probably due to the abundant sequence resources for these two species compared with parasitoid wasps. The low copy number of *Lep*1-like element identified in *N. bombycis* and polydnaviruses might be explained by the low likelihood of fixation and rapid removal of nonessential DNA in their genomes^7^.

### Evidence of horizontal transfer of non-autonomous *Lep*1 *Helitrons*

Traditionally, horizontal transfer has been implied when highly similar TEs have been found in distantly related taxa accompanied by their discontinuous distribution, and such phenomenon could not be explained in terms of vertical inheritance^21^,^22^,^23^. In this study, a patchy taxonomic distribution of *Lep*1 was clearly revealed by database searches. While *Lep*1-like elements were detected in five non-lepidopteran insect species including two aphids (*A. pisum* and *A. gossypii*, Hemiptera), one beetle (*A. glabripennis*, Coleoptera), and two parasitoid wasps (*C. vestalis* and *C. floridanum*, Hymenoptera), no significant hits were observed in the genomes of *R. prolixus* (Hemiptera), *T. castaneum* (Coleoptera), *N. bombycis* and *A. mellifera*, as well as six ants (Hymenoptera). Remarkably, *Lep*1-like elements were also detected in one intracelluar microsporidia parasite, *N. bombycis*, and two bracoviruses which are symbiotically associated with hymenopteran parasitic wasps. In many cases, the sequence identity of the *Lep*1 *Helitrons* is exceptionally high compared with the divergence of the hosts. For example, hymenopteran *CvLep*1_1 showed 90% identity with lepidopteran *Lep*1 consensus sequence, which diverged 325 million years ago (http://www.timetree.org/)^24^, and *CsBVLep*1_1 and *NbLep*1_1 showed 94% and 93% identity with *Lep*1, respectively.

**Figure 1:**
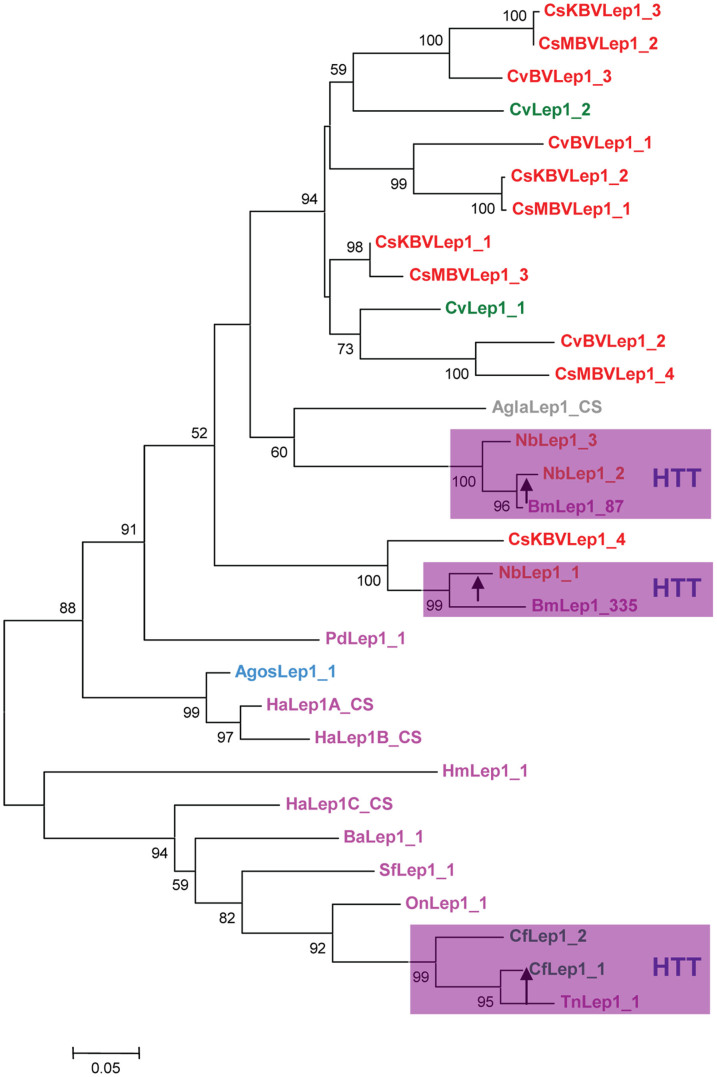
Phylogenetic relationships among *Lep*1-like elements in non-lepidopteran species and representative lepidopteran insect species. The Neighbor-joining tree was generated in MEGA5 with 1000 bootstrapping. Bootstrap values below 50% are not shown. *Lep1*-like elements in non-lepidopteran species were derived from database homology searches, and the abbreviations and GenBank entries were described in Table 1. Consensus sequences of *HaLep*1 lineage A (HaLep1A CS), *HaLep*1 lineage B (HaLep1B CS), *HaLep*1 lineage C (*HaLep*1C CS) and *AglaLep*1 (*AglaLep*1 CS) were derived from multiple sequences alignments in this study. *Trichoplusia ni* *TnLep*1_1 was obtained from database homology searches using *CfLep*1_1 as query, and it's GenBank entry is FF372817. Other representative lepidopteran *Lep*1 elements were derived from Coates et al.^18^, and are obtained from the following GenBank entries: D86623.1 for *Bombyx mori BmLep*1_335, DQ242656.1 for *B. mori* *BmLep*1_87, CR974474 for *Heliconius melpomene HmLep*1_1, AC239123 for *Bicyclus anynana BaLep*1_1, FP340414 for *Spodoptera frugiperda SfLep*1_1, EU532470 for *Ostrinia nubilalis OnLep*1_1, FM995623 for *Papilio dardanus PdLep*1_1. Taxa showing *Lep*1 are colored taxonomically, with lepidopteran insects in purple, Hymenoptera wasps in green, Hemiptera aphids in light blue, Coleoptera beetle in gray, bracoviruses in red, and *Nosema bombycis* in orange.

In an effort to investigate the relationships within *Lep*1 more closely, we reconstructed phylogenetic trees that focuses on these elements and representative lepidopteran *Lep*1 elements. The results obtained with NJ and ML methods were mostly congruent. We chose to present the topologies obtained by NJ method (Fig. 1). The ML tree is provided in Figure S5. The result indicates the existence of two major clades (Fig. 1). The largest clade comprised *Lep*1-like sequences from bracoviruses, *N. bombycis*, *C. vestalis*, *A. glabripennis*, *A. gossypii*, and representative *Lep*1 elements from *B. mori* (*BmLep*1_335 and *BmLep*1_87), *Papilio dardanus* (*PdLep*1_1), and *H. armigera* (HaLep1A and HaLep1B). Inside this clade, two subclades formed by *CsKBVLep*1_4, *NbLep*1_1, *BmLep*1_335, and *A. gossypii* *AgosLep*1_1, *HaLep*1A and *HaLep*1B, respectively, were strongly supported (100% and 99%), and *CvLep*1_1, *CsMBVLep*1_4, and *CvBVLep*1_2 were clustered together, with a bootstrap value of 73%. In the second clade, the *Lep*1-like sequences from *C. floridanum* (*CfLep*1_1 and *CfLep*1_2) were clustered with *Trichoplusia ni* *TnLep*1_1 (FF372817), with a significant bootstrap value of 99%. These results suggested the occurrence of HT and that multiple mechanisms may underlie the horizontal spread of *Lep*1.

**Figure 2:**
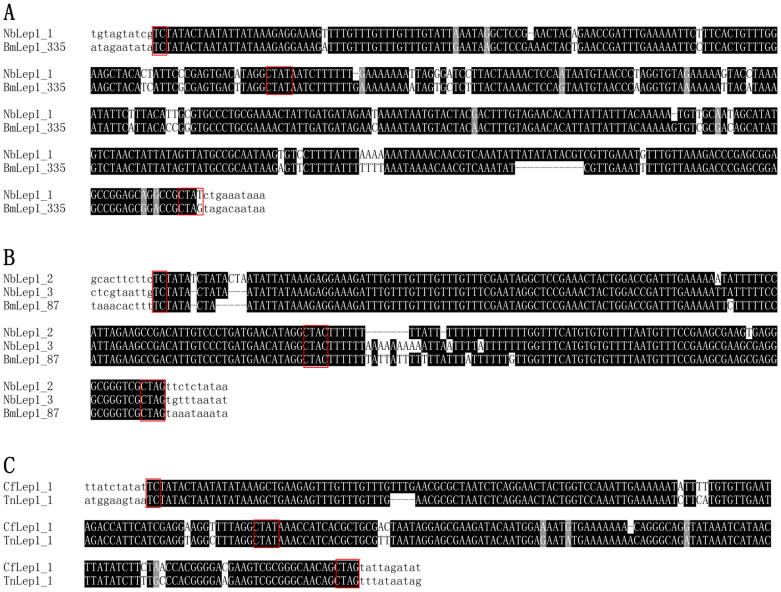
Alignments of selected sequences from GenBank entries sharing high identity with *Nosema bombycis* *NbLep*1_1 (A), *NbLep*1_2 and *NbLep*1_3 (B) and *Copidosoma floridanum* *CfLep*1 (C). Nucleotides shaded in black are conserved across sequences. Typical structural features of the *Lep1* elements including characteristic 5′-TC and 3′-CTRY nucleotide termini as well as CTRR motif at the 3′ end of acquired sequence were boxed. Abbreviations and GenBank entries for these elements are described in Figure 1.

While the inherent abilities of TEs to replicate and integrate into the host genome undoubtedly facilitate HT between organisms, the precise mechanisms underling HTT remain largely mysterious. Several hypotheses have been proposed to explain how TEs might be transferred between eukaryotic hosts. For example, TEs can putatively explore events like parasite mediated transfers from one host to another^25^, as in the case of the *mariner* element transferred between the braconid parasitoid wasp, *Ascogaster reticulatus*, and its moth host, the smaller tea tortrix, *Adoxophyes honmai*^26^. The little interspecific sequence similarity of acquired sequences at 3′ end makes *Lep*1 a good candidate for the study of HTT mechanisms. In this study, the identification of *Lep*1 *Helitrons* in *C. floridanum* and *N. bombycis* as well as their lepidopteran host insects is of particular interest. *C. floridanum* is a polyembryonic encyrtid that parasitizes the egg stage of *T. ni* and related moth species^27^,^28^. The *N. bombycis* is well known as the causal agent of microsporidun disease pébrine of silkworm larvae, *B. mori*^29^. Sequence comparison showed that, across the entire length of the elements, *CfLep*1_1 showed 94% identity with *TnLep*1_1, *NbLep*1_1 showed 91% identity with *BmLep*1_335, and *NbLep*1_2 and *NbLep*1_3 showed 98% and 94% identity with *BmLep*1_87, respectively. Specifically, the acquired sequences of both *NbLep1* and *CfLep*1_1 showed over 90% identity with their lepidopteran host *Lep*1 elements (Fig. 2). Thus, our study provides evidence of the occurrence of HTT facilitated by host-parasite interactions.

### Putative directions of horizontal transfer of *Lep*1 *Helitrons*

**Figure 3:**
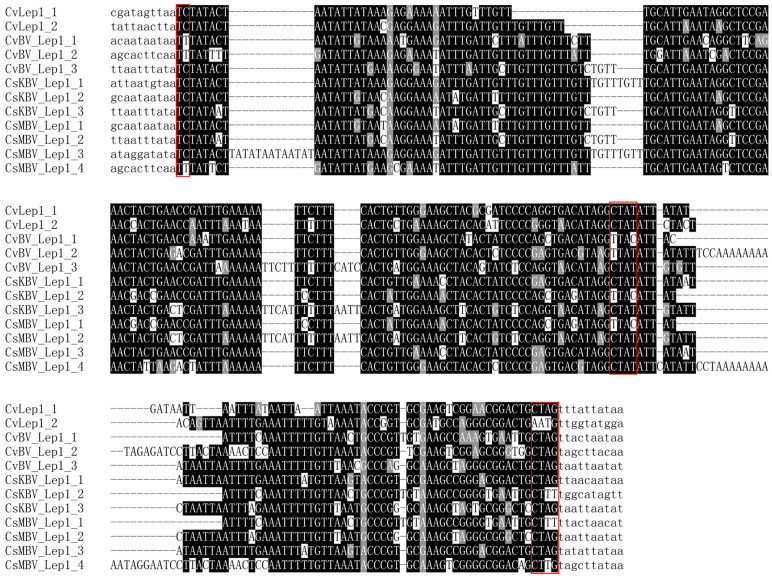
Alignments of bracovirus and parasitoid wasp *Lep*1-like elements. Nucleotides shaded in black are conserved across sequences. Typical structural features of the *Lep*1 elements including characteristic 5′-TC and 3′-CTRY nucleotide termini as well as CTRR motif at the 3′ end of acquired sequence were boxed. Abbreviations and GenBank entries for these elements are described in Table1.

The Polydnaviruses display an obligatory relationship with endoparasitoid wasps belonging to the Braconidae family and Ichneumonid family, and have been proposed to be potential vectors for the delivery of TEs among species^30^. During the past few years, there have been several reports of TE-like sequences in the genomes of Polydnaviruses^31^,^32^,^33^,^34^,^35^. In this study, *Lep*1-like sequences were identified in *C. vestalis* bracovirus (CvBV), and *C. sesamiae* bracovirus from Kitale (CsKBV) and Mombasa (CsMBV) strains. These results suggested that Polydnaviruses might be important vectors of HT of *Lep*1 *Helitrons*. Interestingly, *Lep*1-like sequences were also identified in the parasitoid wasp, *C. vestalis*. Considering the widespread distribution of *Lep*1-like sequences in lepidopteran species, it is reasonable to propose that *Lep*1 *Helitrons* were transferred from lepidopteran hosts to parasitoid wasps using polydnaviruses to mediate the actual transfer of TE DNA between cells. However, the acquired sequences of *CvLep*1 and *CvBvLep*1 showed only moderate similarity (72% between *CvLep*1_2 and *CvBvLep*1_3) (Fig. 3). This is possibly because of the current limited availability of *C. vestalis* sequence. *C. vestali* is larval parasitoid of the diamondback moth, *Plutella xylostella* (Lepidoptera: Plutellidae). However, we also did not find sequences similar to acquired sequences of *CvLep*1and *CvBvLep*1 in the genome database of *P. xylostella* (http://iae.fafu.edu.cn/DBM/). Because in some cases, parasitoids are likely to oviposit within marginal (or even completely unsuitable) hosts in the laboratory or field, even if suitable hosts are present^36^, and *C. vestalis* has been reared from several species belonging to different lepidopteran families^37^, we propose that *CvLep1* identified in this study may be transferred from other lepidopteran host to *C. vestalis*. This hypothesis could be partly supported by the fact observed in this study: the acquired sequence of *CsKBVLep*1_4 showed 90% similarity with *BmLep*1_335, suggesting that *C. sesamiae* might have oviposited within *B. mori* (Fig. S6). Alternatively, considering that the Braconidae wasps form a monophyletic assemblage named the microgastroid complex, which evolved 100 million years ago, and BVs evolved from the interaction between the common ancestor of microgastroids and a single ancestral virus^38^,^39^, the lepidopteran *Lep*1 might repeatedly invade into the common ancestor of BV, and then horizontally transfer to *Cotesia* parasitoids. This hypothesis could be supported by the facts observed in this study: the acquired sequence of *CvBvLep*1_1 and *CvBvLep*1_3 showed 88% similarity with *CsKBVLep*1_2 and *CsMBVLep*1_1, and *CsKBVLep*1_3 and *CsMBVLep*1_2, respectively (Fig. 3), suggesting that *Lep*1-like element might insert into the common ancestor genome of these viruses. Additional experiments and taxon sampling are necessary to further determine the direction and frequency of HT of *Lep*1 *Helitrons*.

### Other putative mechanisms underlying horizontal transfer of *Lep*1 *Helitrons*

While our results indicate the role of host-parasite interactions in HT of *Lep*1, the presence of *Lep*1-like elements in *A. glabripennis* and *A. pisum* as well as *A. gossypii* is somewhat intriguing. Notably, a recent study also showed the occurrence of horizontal transfer of short interspersed nuclear elements (*HaSE2*) between Heliothine species and *A. gossypii*^40^. It has been proposed that mechanisms of HT include insect-associated facultative symbionts such as genera *Wolbachia*, *Rickettsia, Cardinium, Arsenophonus*, and *Sodalis*^41^,^42^,^43^,^44^,^45^. In addition to the possibility of HT through facultative symbionts, the *Lep*1-like elements identified in *N. bombycis* in this study suggested that the intracellular microsporidia parasite is also a potential vector for HT. It is reported that *Wolbachia* infect at least 20% of all insect species including aphids^46^,^47^,^48^, and apart from the domesticated silkworms, *N. bombycis* can also infect various lepidopteran insects^49^,^50^,^51^, indicative of their broad hosts range. Additionally, a previous study showed that *A. glabripennis* could be infected by microsporidia parasite, *Nosema glabripennis*^52^. Thus, we proposed that facultative symbionts including *Wolbachia* and obligate intracellular microsporidia parasites might play a role in the HT of *Lep*1-like elements in *A. glabripennis* and *A. pisum* as well as *A. gossypii*. More widespread sequencing would be required to find exact vectors that would facilitate the HT of *Lep*1 *Helitrons* in these species.

## Methods

### DNA extraction and genome walking

A previous study has shown that TEs were enriched within or in close proximity to xenobiotic-metabolizing cytochrome P450 genes in *H. zea*^53^. To isolate TEs in *H. armigera*, we performed genome walking to obtain the 5′-flanking sequence of an insecticide resistance-associated cytochrome P450 gene, *CYP6AE12*, in *H. armigera*^54^. Genomic DNA was isolated from individual third instar larva, using the procedure described by Wang et al^55^. Gene-specific primers based on the known sequence of the cDNA (accession number: DQ256407) and four general primers provided by the Genome Walking Kit (TaKaRa, Dalian, China) were used for every genome walking. PCR products were cloned into pGEM-T Easy vector (Promega, Madison, WI, USA) and sequenced.

### Database search strategy

Database searches were performed and comprise four steps. Firstly, the *Lep*1-like element (named as *HaLep1_1*) identified in the 5′-flanking sequence of the *H. armigera* P450 gene, *CYP6AE12*, was compared with NCBI *H. armigera* nucleotide collection (nr/nt) databases with Blastn (www.ncbi.nlm.gov/cgibin/BLAST), sequences of high homology as well as 500 bp upstream and downstream flanking regions were extracted and analyzed for hallmarks of *Lep*1 *Helitrons* such as characteristic 5′-TC and 3′-CTRY nucleotide termini as well as CTRR motif at the 3′ end of acquired sequence. Secondly, nucleotide (*nr/nt*) and EST (*est_others*) collections were searched using *HaLep*1_1 as query to detect sequences with high identity with *HaLep*1_1 in lepidopteran species other than *H. armigera*. Thirdly, the 134-bp lepidopteran-specific common sequence 3 (LSCS3, *Lep*1) was searched against non-lepidopteran insect genome sequences, including BeetleBase (http://beetlebase.org/), AphidBase (http://www.aphidbase.com/aphidbase/), NasoniaBase (http://hymenopteragenome.org/nasonia/), BeeBase (http://hymenopteragenome.org/beebase/), vectorbase (https://www.vectorbase.org), and Ant Genomes Portal (http://hymenopteragenome.org/ant_genomes/). Finally, The 134 bp Lep1 consensus sequence was compared with NCBI non-lepidopteran databases with Blastn, including the whole genome shotgun, nucleotide collection (nr/nt), genome survey sequences, high throughput genomic sequences, and expressed sequence tag databases. Hits that were ≥70% identical to the query over >100 bp were examined and, when possible, full-length *Lep*1-like elements were manually extracted. These elements were used as queries to find additional related *Lep*1 *Helitrons*, the resulting hits were examined, and full-length elements were extracted.

### Assessing polymorphism

In *H. armigera*, using one pair of primers flanking the insertion site, *HaLep*1_1 insertion polymorphism was assessed by performing a PCR survey, which yielded products of different sizes in *HaLep*1_1 insertion individual (about 700 bp) and non-insertion individual (about 500 bp). To further illustrate the mobility of other *HaLep*1 elements, the insertion polymorphisms were also assessed by homology searches. Briefly, paralogous or orthologous sites not containing a *HaLep*1 insertion (empty sites) were identified by homology searches utilizing Blastn with a query constructed from the sequences directly flanking the insertion site. The chimeric query sequence (about 200 bp in length) was created by extracting both the flanking sequence upstream from the element insertion (about 100 bp) and the flanking sequence downstream from the element insertion (about 100 bp).

### Sequence analysis

Multiple sequence alignments were performed using ClustalW^56^ with default settings. Neighbor-joining (NJ) and maximum likelihood (ML, using the Tamura-Nei model) phylogenetic trees were constructed using Mega 5^57^. The reliability of the NJ and ML tree topology was statistically evaluated by bootstrap analysis with 1000 replicates. To detect putative cross-species contamination during DNA sequencing, 10 kb sequences in each direction (upstream and downstream) of each representative non-lepidopteran *Lep1* insertion were extracted from the BAC clone sequences and used to search against the non-redundant databases using the NCBI server with Blastx (www.ncbi.nlm.gov/cgibin/BLAST).

## Author Contributions

Conceived and designed the experiments: J.W. Performed the experiments: J.W., X.G., J.G. Analyzed the data: J.W., X.G., J.G., F.L. Wrote the paper: J.W., F.L.

## Supplementary Material

Supplementary Information Supporting information

## Figures and Tables

**Table 1:** Full length putative *Lep*1-like elements identified in non-lepidopteran species. The 134 bp *Lep*1 consensus sequence was used as a query. Sequence similarity with *Lep*1 was calculated excluding indels

Name	Genus species	GenBank accession	location	Size (bp)	% sim.
CvLep1_1	Cotesia vestalis	GAKG01025082	774–963	190	90
CvLep1_2	Cotesia vestalis	GAKG01005573	371–171	201	86
CvBVLep1_1	Cotesia vestalis BV	HQ009543	29755–29864	196	83
CvBVLep1_2	Cotesia vestalis BV	HQ009537	7652–7557	224	83
		DQ075354	7743–7648		
CvBVLep1_3	Cotesia vestalis BV	EF067323	7651–7814	218	88
CsKBVLep1_1	Cotesia sesamiae KBV	EF710635	103544–103433	213	94
CsKBVLep1_2	Cotesia sesamiae KBV	EF710634	3978–3860	196	84
		EF710628	66996–66890		
		HF562925	4899–5005		
CsKBVLep1_3	Cotesia sesamiae KBV	EF710633	63741–63590	218	83
CsKBVLep1_4	Cotesia sesamiae KBV	EF710634	3978–3860	344	90
		EF710633	11656–11767		
CsMBVLep1_1	Cotesia sesamiae MBV	EF710642	5160–5066	196	84
		EF710638	98398–98504		
CsMBVLep1_2	Cotesia sesamiae MBV	EF710641	36832–36943	218	83
CsMBVLep1_3	Cotesia sesamiae MBV	EF710642	88205–88310	226	94
CsMBVLep1_4	Cotesia sesamiae MBV	EF710640	4706–4846	227	82
NbLep1_1	Nosema bombycis	ACJZ01002694	334–778	445	93
NbLep1_2	Nosema bombycis	ACJZ01001444	842–635	208	83
NbLep1_3	Nosema bombycis	ACJZ01001453	267–484	218	84
CfLep1_1	Copidosoma floridanum	JI831644	358–106	253	75
CfLep1_2	Copidosoma floridanum	JI839208	363–128	236	69
AgosLep1_1	Aphis gossypii	GW506388	153–362	210	86
