# Supplementary material for: Evidence of horizontal transfer of non-autonomous Lep1 Helitrons facilitated by host-parasite interactions
Source: Sci Rep. 2014 May 30;4:5119. doi: 10.1038/srep05119 (PMC4038834; doi:10.1038/srep05119)
Supplement: Supplementary Information — Supporting information [file srep05119-s1.pdf]

Evidence of horizontal transfer of non-autonomous *Lep*1 *Helitrons* facilitated by host-parasite interactions

Xuezhu Guo[1]， Jingkun Gao[1], Fei Li[2] & Jianjun Wang[1]

[1]College of Horticulture and Plant Protection, Yangzhou University, Yangzhou, 225009, China;

[2]College of Plant Protection, Nanjing Agricultural University, Nanjing, 210095, China

Supplementary Figure Legends

Figure S1. Alignments of full length *HaLep*1 elements identified in this study. Typical structural features of the *Lep1* elements including characteristic 5'-TC and 3'-CTRY nucleotide termini as well as CTRR motif at the 3' end of acquired sequence were boxed. Nucleotides shaded in black are conserved across sequences. All elements excluding *HaLep1_1* were derived from database homology searches, and the GenBank entries were described in Table S1.

Figure S2. Phylogenetic relationships among full length *HaLep1* elements in *Helicoverpa armigera*. The Neighbor-joining tree was generated in MEGA5 with 1000 bootstrapping. Bootstrap values below 50% are not shown. All elements excluding *HaLep1_1* were derived from database homology searches, and the GenBank entries were described in Table S1.

Figure S3. Alignments of representative *Helicoverp zea* (EF152213, EF152207, HQ840515) and *Heliothis virescens* (GT133743, GR964554, GT133396) sequences from GenBank entries sharing high identity with *HaLep*1_1. Typical structural features of the *Lep*1 elements including characteristic 5'-TC and 3'-CTRY nucleotide termini as well as CTRR motif at the 3' end of acquired sequence were boxed. Nucleotides shaded in black are conserved across sequences.

Figure S4. Insertion polymorphism of *HaLep*1_1 (A), *HaLep*1_20 (B), and *HaLep*1_8 (C) elements at paralogous sites of *Helicoverpa armigera* or orthologous sites of *Helicoverpa zea*. Typical structural features of the *Lep*1 elements including characteristic 5'-TC and 3'-CTRY nucleotide termini as well as CTRR motif at the 3' end of acquired sequence were boxed.

Figure S5. Phylogenetic analysis of *Lep*1-like elements in non-lepidopteran species and representative lepidopteran insect species using maximum likelihood method.

Figure S6. Alignments of bracovirus *CsKBV_Lep*1_4 and *Bombyx mori BmLep*1_335

elements. Typical structural features of the *Lep*1 elements including characteristic 5'-TC and 3'-CTRY nucleotide termini as well as CTRR motif at the 3' end of acquired sequence were boxed. Abbreviations and GenBank entries for these elements are described in Table 1.

Table S1. Positions of full length putative *HaLep*1 *Helitrons* within GenBank nr/nt database accessions. Sequence similarity with 134 bp *Lep*1 concensus sequence was calculated excluding indels.

Name	GenBank accession	description	location	Size (bp)	% sim.
HaLep1_1	KJ010530	microsomal cytochrome P450 (CYP6AE12) gene flanking sequence	548-357	192	75
HaLep1_2	FP340431.1	BAC	40010- 39816	195	76
HaLep1_3	FP340432.1	BAC	96619- 96424	196	76
HaLep1_4	FP340432.1	BAC	105945- 106114	170	78
HaLep1_5	FP340436.1	BAC	64381- 64571	191	71
HaLep1_6	FP340422.1	BAC	78094- 77909	186	68
HaLep1_7	AY714875.1	E-cadherin gene	2216-2022	195	75
HaLep1_8	EU327673.1	microsomal cytochrome P450 (CYP9A12) gene	2394-2593	200	88
HaLep1_9	FJ416332.1	microsomal cytochrome P450 (CYP9A17) gene	3047-3246	200	88
HaLep1_10	FO082297.1	BAC	68036- 68217	182	85
HaLep1_11	FO082297.1	BAC	75053- 75242	190	84
HaLep1_12	FP340435.1	BAC	24342- 24143	200	71
HaLep1_13	JX306730.1	esterase mRNA	1881-1688	194	75
HaLep1_14	FP340433.1	BAC	10113- 10308	196	84
HaLep1_15	FP340433.1	BAC	89748- 89554	195	88
HaLep1_16	JQ995292.1	BAC	68026- 68221	196	88
HaLep1_17	FP340434.1	BAC	49161- 49365	205	84
	FP340425.1	BAC	68006-67802		
	FP340421.1	BAC	86789- 86585		
HaLep1_18	FP340430.1	BAC	38976- 38772	205	84
HaLep1_19	FP340434.1	BAC	23715- 23516	200	84
	FP340425.1	BAC	93434-93633		
	FP340421.1	BAC	112217- 112416		
HaLep1_20	FP340425.1	BAC	4919-4708	212	89
	FP340421.1	BAC	23702- 23487	216	88
HaLep1_21	FP340429.1	BAC	11885- 12080	196	87
HaLep1_22	JQ995291.1	cytochrome P450 337B2v2 and cytochrome P450 337B1v1 gene	9985-9785	201	83

Table S2. Top-scoring BLAST hits in lepidopteran species using acquired sequences of full length putative *HaLep*1 *Helitrons* as queries. Sequence similarity was calculated excluding indels.

Name	Size (bp)	Nr/nt database			EST database		
		species	GenBank Accession	% sim.	species	GenBank Accession	% sim.
HaLep1_1	62	Helicoverpa zea	EF152213.1	97	Heliothis virescens	GT203206.1	94
HaLep1_2	63	Helicoverpa zea	EF152213.1	95	Heliothis virescens	GT203206.1	92
HaLep1_3	63	Helicoverpa zea	EF152213.1	95	Heliothis virescens	GT203206.1	92
HaLep1_4	62	Helicoverpa zea	EF152213.1	95	Heliothis virescens	GT203206.1	92
HaLep1_5	63	Helicoverpa zea	EF152213.1	93	Heliothis virescens	GT203206.1	89
HaLep1_6	63	Helicoverpa zea	EF152213.1	97	Heliothis virescens	GT203206.1	94
HaLep1_7	59	Helicoverpa zea	EF152207.1	98	Heliothis virescens	GT210228.1	92
HaLep1_8	64	Helicoverpa zea	M80588.1	92	Heliothis virescens	GT135677.2	94
HaLep1_9	64	Helicoverpa zea	M80588.1	92	Heliothis virescens	GT135677.2	94
HaLep1_10	54	Helicoverpa zea	M80588.1	85	Heliothis virescens	JK135832.1	96
HaLep1_11	64	Helicoverpa zea	M80588.1	85	Heliothis virescens	GT135677.2	89
HaLep1_12	64	Helicoverpa zea	EF152207.1	95	Heliothis virescens	GT210228.1	90
HaLep1_13	64	Helicoverpa zea	EF152207.1	82	Heliothis virescens	GT206484.1	78
HaLep1_14	64	Helicoverpa zea	M80588.1	87	Heliothis virescens	GT135677.2	89
HaLep1_15	64	Helicoverpa zea	M80588.1	90	Heliothis virescens	GT135677.2	91
HaLep1_16	64	Helicoverpa zea	M80588.1	90	Heliothis virescens	GT135677.2	91
HaLep1_17	64	Helicoverpa zea	M80588.1	87	Heliothis virescens	GT135677.2	91
HaLep1_18	64	Helicoverpa zea	M80588.1	87	Heliothis virescens	GT135677.2	91
HaLep1_19	64	Helicoverpa zea	M80588.1	89	Heliothis virescens	GT131399.2	89
HaLep1_20	64	Helicoverpa zea	M80588.1	89	Heliothis virescens	GT055003.1	92
HaLep1_21	64	Helicoverpa zea	M80588.1	89	Heliothis virescens	GT135677.2	91
HaLep1_22	64	Helicoverpa zea	M80588.1	87	Heliothis virescens	GT131399.2	92

Table S3. Putative *Lep*1-like elements identified in the genome database of *Acyrthosiphon pisum* (AphidBase 2.1). The 134 bp *Lep*1 concensus sequence was used as a query, and "hits" were filtered for 70% homology over ≥100 bp.

Name	Scaffold	% sim	Aligned length	Query start	Query stop	"Hit" start	"Hit" stop	*E*-value	Bit score
AP_Lep1_1	EQ121671.1	87	126	9	134	2166	2041	2e-37	157
AP_Lep1_2	EQ121807.1	84	134	1	133	29624	29491	1e-33	144
AP_Lep1_3	EQ120321.1	85	124	2	125	13387	13264	1e-33	144
AP_Lep1_4	EQ126612.1	84	131	2	132	13541	13411	4e-33	143
AP_Lep1_5	EQ117863.1	85	133	1	133	24998	25128	4e-33	143
AP_Lep1_6	EQ117863.1	81	140	1	133	2315	2176	6e-26	119
AP_Lep1_7	EQ112278.1	83	131	2	132	256081	256211	5e-32	139
AP_Lep1_8	EQ126085.1	84	133	1	133	5248	5378	2e-31	137
AP_Lep1_9	EQ126085.1	78	108	1	105	38946	38839	2e-13	77.6
AP_Lep1_10	EQ123292.1	83	130	4	133	2674	2545	2e-31	137
AP_Lep1_11	EQ123292.1	84	129	1	129	11047	10921	2e-30	133
AP_Lep1_12	EQ123292.1	82	130	4	133	5217	5088	8e-30	132
AP_Lep1_13	EQ114880.1	84	125	9	133	31055	30931	2e-31	137
AP_Lep1_14	EQ120778.1	83	132	1	132	151741	151872	7e-31	135
AP_Lep1_15	EQ119732.1	83	134	1	133	245	112	7e-31	135
AP_Lep1_16	EQ126005.1	83	136	1	133	6896	6761	2e-30	133
AP_Lep1_17	EQ121771.1	82	135	1	133	437872	438006	8e-30	132
AP_Lep1_18	EQ121771.1	77	132	3	133	437550	437680	3e-17	90.3
AP_Lep1_19	EQ116775.1	83	125	9	133	177549	177673	8e-30	132
AP_Lep1_20	EQ128583.1	83	134	1	133	9817	9917	3e-29	130
AP_Lep1_21	EQ128017.1	82	134	1	132	30913	31046	3e-29	130
AP_Lep1_22	EQ125783.1	83	137	1	133	31773	31638	3e-29	130
AP_Lep1_23	EQ117504.1	86	109	1	109	178	70	3e-29	137
AP_Lep1_24	EQ111315.1	84	134	1	133	38524	38657	3e-29	130
AP_Lep1_25	EQ120795.1	83	128	6	133	3030	2904	1e-28	128
AP_Lep1_26	EQ120795.1	80	132	1	132	102326	102195	5e-27	123
AP_Lep1_27	EQ112817.1	82	128	1	128	16625	16498	1e-28	128
AP_Lep1_28	EQ124027.1	81	134	1	133	16559	16426	4e-28	126
AP_Lep1_29	EQ121809.1	83	132	2	132	63711	63841	4e-28	126
AP_Lep1_30	EQ121277.1	81	132	1	132	29922	30053	4e-28	126
AP_Lep1_31	EQ119276.1	81	134	1	133	15813	15680	4e-28	126
AP_Lep1_32	EQ113439.1	81	132	1	132	8710	8579	4e-28	126
AP_Lep1_33	EQ120981.1	83	123	1	123	2894	2773	1e-27	124
AP_Lep1_34	EQ121672.1	87	105	1	105	4653	4756	5e-27	123
AP_Lep1_35	EQ120289.1	83	123	6	126	87885	87763	5e-27	123
AP_Lep1_36	EQ117295.1	74	127	16	133	36596	36722	6e-08	59.4

AP_Lep1_37	EQ112273.1	81	132	1	132	110908	111038	5e-27	123
AP_Lep1_38	EQ111282.1	87	105	1	105	35737	35634	5e-27	123
AP_Lep1_39	EQ122832.1	80	128	1	128	26143	26170	6e-26	119
AP_Lep1_40	EQ122498.1	82	130	6	134	27805	27677	6e-26	119
AP_Lep1_41	EQ120520.1	80	128	2	129	3184	3311	6e-26	119
AP_Lep1_42	EQ115814.1	81	125	9	133	84028	84152	6e-26	119
AP_Lep1_43	EQ128156.1	81	132	2	132	3797	3927	2e-25	117
AP_Lep1_44	EQ127029.1	81	130	6	133	148637	148766	2e-25	117
AP_Lep1_45	EQ125279.1	80	132	1	132	240480	240610	2e-25	117
AP_Lep1_46	EQ124938.1	81	124	9	132	10468	10345	2e-25	117
AP_Lep1_47	EQ123980.1	82	124	4	127	5252	5131	2e-25	117
AP_Lep1_48	EQ117422.1	80	129	1	129	4959	5087	2e-25	117
AP_Lep1_49	EQ111442.1	81	124	1	124	27185	27062	2e-25	117
AP_Lep1_50	EQ112802.1	79	133	1	133	48081	47879	7e-25	115
AP_Lep1_51	EQ110838.1	81	131	4	133	77441	77312	7e-25	115
AP_Lep1_52	EQ128634.1	74	105	5	109	19982	20084	6e-08	59.4
AP_Lep1_53	EQ125405.1	80	134	1	133	14977	14845	9e-24	112
AP_Lep1_54	EQ110923.1	79	125	9	133	55549	55425	3e-23	110
AP_Lep1_55	EQ124786.1	73	100	1	100	56943	56844	6e-08	59.4
AP_Lep1_56	EQ128618.1	79	127	6	132	4741	4615	1e-22	108
AP_Lep1_57	EQ126112.1	72	135	1	133	20398	20531	6e-08	59.4
AP_Lep1_58	EQ128067.1	75	102	1	100	24238	24137	6e-08	59.4
AP_Lep1_59	EQ113816.1	84	104	2	105	1903	2005	1e-22	108
AP_Lep1_60	EQ111330.1	81	124	4	127	16137	16016	1e-22	108
AP_Lep1_61	EQ115875.1	74	100	1	100	16844	16746	6e-08	59.4
AP_Lep1_62	EQ120330.1	76	109	4	112	33264	33165	3e-06	54.0
AP_Lep1_63	EQ126059.1	79	141	1	133	7161	7021	4e-22	106
AP_Lep1_64	EQ121344.1	82	110	25	133	18414	18305	1e-21	104
AP_Lep1_65	EQ112414.1	71	133	1	132	17144	17013	2e-08	61.2
AP_Lep1_66	EQ115294.1	71	133	1	132	29669	29538	2e-08	61.2
AP_Lep1_67	EQ111302.1	80	135	1	133	35219	35351	1e-21	104
AP_Lep1_68	EQ117283.1	71	133	1	132	52730	52599	2e-08	61.2
AP_Lep1_69	EQ118791.1	71	133	1	132	5104	4973	2e-08	61.2
AP_Lep1_70	EQ119975.1	79	134	1	133	14403	14272	5e-21	103
AP_Lep1_71	EQ121841.1	73	127	12	134	8465	8340	2e-08	61.2
AP_Lep1_72	EQ120279.1	73	128	8	134	31297	31172	2e-08	61.2
AP_Lep1_73	EQ119794.1	71	133	1	132	43227	43358	2e-08	61.2
AP_Lep1_74	EQ123422.1	77	132	3	133	10925	11096	6e-20	99.4
AP_Lep1_75	EQ114336.1	82	102	4	105	1413	1513	6e-20	99.4
AP_Lep1_76	EQ114320.1	77	132	1	132	15396	15527	6e-20	99.4
AP_Lep1_77	EQ111794.1	78	122	9	130	97576	97697	6e-20	99.4
AP_Lep1_78	EQ111786.1	75	101	1	100	95082	94984	2e-07	57.6
AP_Lep1_79	EQ122846.1	71	133	1	132	50968	50837	2e-08	61.2
AP_Lep1_80	EQ124414.1	78	134	1	133	14836	14965	2e-19	97.6

AP_Lep1_81	EQ118785.1	79	140	1	133	42613	42474	2e-19	97.6
AP_Lep1_82	EQ115073.1	81	103	3	105	12624	12726	2e-19	97.6
AP_Lep1_83	EQ124367.1	71	133	1	132	68128	67996	2e-08	61.2
AP_Lep1_84	EQ128535.1	78	124	2	123	103743	103621	3e-18	94
AP_Lep1_85	EQ127050.1	80	132	2	132	16928	19807	3e-18	94
AP_Lep1_86	EQ124523.1	78	125	9	129	1323	119	3e-18	94
AP_Lep1_87	EQ127018.1	79	131	1	122	79181	79051	9e-18	92.1
AP_Lep1_88	EQ127018.1	78	131	1	122	75149	75279	1e-16	88.5
AP_Lep1_89	EQ120317.1	81	105	1	105	5076	5178	9e-18	92.1
AP_Lep1_90	EQ113362.1	77	133	1	132	5747	5616	9e-18	92.1
AP_Lep1_91	EQ124619.1	71	133	1	132	19374	19505	2e-08	61.2
AP_Lep1_92	EQ132906.1	77	132	3	133	350	220	3e-17	90.3
AP_Lep1_93	EQ117841.1	77	133	1	132	30371	30235	3e-17	90.3
AP_Lep1_94	EQ116279.1	79	104	29	132	87277	87380	3e-17	90.3
AP_Lep1_95	EQ114788.1	78	142	2	133	44263	44123	3e-17	90.3
AP_Lep1_96	EQ112293.1	79	116	14	129	89923	90032	3e-17	90.3
AP_Lep1_97	EQ125990.1	71	133	1	132	802	934	2e-08	61.2
AP_Lep1_98	EQ121327.1	76	100	1	100	18186	18091	5e-09	63.1
AP_Lep1_99	EQ114318.1	80	113	6	111	20671	20560	4e-16	86.7
AP_Lep1_100	EQ127501.1	78	109	25	133	404708	404601	1e-15	84.9
AP_Lep1_101	EQ125985.1	77	106	27	132	17323	17428	1e-15	84.9
AP_Lep1_102	EQ127853.1	71	135	1	133	2762	2895	8e-07	55.8
AP_Lep1_103	EQ128009.1	72	134	1	132	24376	24243	5e-09	63.1
AP_Lep1_104	EQ113793.1	79	108	1	108	55715	55818	1e-15	84.9
AP_Lep1_105	EQ127558.1	79	103	1	103	7583	7684	5e-15	83.1
AP_Lep1_106	EQ123782.1	76	133	3	131	138571	138703	5e-15	83.1
AP_Lep1_107	EQ122883.1	77	132	9	133	11879	12010	5e-15	83.1
AP_Lep1_108	EQ128435.1	73	127	3	122	2869	2995	5e-09	63.1
AP_Lep1_109	EQ121800.1	73	132	1	132	35480	35349	2e-14	81.2
AP_Lep1_110	EQ111137.1	75	100	1	100	1253	1351	1e-09	64.9
AP_Lep1_111	EQ122273.1	73	104	9	112	572120	572222	2e-07	57.6
AP_Lep1_112	EQ114773.1	75	100	1	100	129338	129240	1e-09	64.9
AP_Lep1_113	EQ128504.1	72	134	1	132	75878	76010	2e-07	57.6
AP_Lep1_114	EQ121795.1	72	130	1	129	55115	55243	1e-09	64.9
AP_Lep1_115	EQ126550.1	72	133	1	132	6790	6659	1e-09	64.9
AP_Lep1_116	EQ125419.1	71	110	1	110	4040	4149	8e-07	55.8
AP_Lep1_117	EQ116885.1	71	133	1	132	6363	6232	8e-07	55.8
AP_Lep1_118	EQ118856.1	73	110	1	109	28	136	8e-07	55.8
AP_Lep1_119	EQ117361.1	74	141	1	133	55576	55716	3e-12	74.0
AP_Lep1_120	EQ115421.1	76	100	11	110	1362	1461	3e-12	74.0
AP_Lep1_121	EQ126146.1	73	132	1	132	14779	14649	1e-11	72.1
AP_Lep1_122	EQ112774.1	75	101	1	100	252267	252167	4e-10	66.7
AP_Lep1_123	EQ118900.1	75	130	2	126	3647	3774	4e-10	66.7
AP_Lep1_124	EQ125271.1	76	109	1	108	14712	14819	1e-11	72.1

AP_Lep1_125	EQ115859.1	76	102	4	105	11071	11171	1e-11	72.1
AP_Lep1_126	EQ123783.1	72	119	16	134	53555	53437	4e-10	66.7
AP_Lep1_127	EQ126057.1	73	133	2	134	32107	32237	3e-11	70.3
AP_Lep1_128	EQ120383.1	77	103	1	102	1759	1860	3e-11	70.3
AP_Lep1_129	EQ119772.1	72	133	1	132	195440	195572	3e-11	70.3
AP_Lep1_130	EQ117828.1	73	133	1	132	48146	48277	3e-11	70.3
AP_Lep1_131	EQ115844.1	73	133	1	132	37531	37400	3e-11	70.3
AP_Lep1_132	EQ117388.1	73	135	1	134	827	960	1e-10	68.5
AP_Lep1_133	EQ128008.1	73	135	1	132	1800	1934	1e-10	68.5
AP_Lep1_134	EQ127059.1	77	103	2	100	4114	4216	1e-10	68.5
AP_Lep1_135	EQ123366.1	73	132	1	132	923	793	1e-10	68.5
AP_Lep1_136	EQ126015.1	73	130	4	132	72104	72232	1e-10	68.5
AP_Lep1_137	EQ121280.1	73	136	1	134	116683	116553	8e-07	55.8
AP_Lep1_138	EQ125290.1	76	102	4	105	973	1072	1e-10	68.5

Table S4. Full length putative *Lep*1-like elements identified in whole genome shotgun database of *Anoplophora glabripennis*. The 134 bp *Lep*1 concensus sequence was used as a query. Sequence similarity with *AglaLep*1 concensus sequence was calculated including/excluding indels.

Name	GenBank accession	Insertion site	Location	Size (bp)	% sim.
AglaLep1_0001	AQHT01005625.1	A/T	957-1161	205	97/99
AglaLep1_0002	AQHT01085866.1	A/T	18428-18224	205	97/99
AglaLep1_0003	AQHT01011904.1	A/T	18478-18274	205	97/99
AglaLep1_0004	AQHT01005409.1	A/T	738-534	205	97/99
AglaLep1_0005	AQHT01027613.1	A/T	4085-4293	205	100/100
AglaLep1_0006	AQHT01017642.1	T/T	1575-1783	209	99/99
AglaLep1_0007	AQHT01074099.1	C/T	14356-14560	205	97/99
AglaLep1_0008	AQHT01056201.1	A/T	14557-14761	205	97/99
AglaLep1_0009	AQHT01040471.1	A/T	7924-7720	205	98/99
AglaLep1_0010	AQHT01057332.1	A/T	13671-13464	209	99/99
AglaLep1_0011	AQHT01051219.1	A/T	34508-34300	209	98/98
AglaLep1_0012	AQHT01022591.1	A/T	1434-1226	209	99/99
AglaLep1_0013	AQHT01021193.1	A/T	15650-15442	209	99/99
AglaLep1_0014	AQHT01015623.1	A/T	19125-18917	209	99/99
AglaLep1_0015	AQHT01007347.1	A/T	12767-12559	209	99/99
AglaLep1_0016	AQHT01007162.1	A/T	850-1058	209	99/99
AglaLep1_0017	AQHT01022591.1	A/T	1434-1226	209	99/99
AglaLep1_0018	AQHT01014629.1	A/T	5065-5269	205	96/98
AglaLep1_0019	AQHT01008721.1	A/T	47774-47973	205	97/99
AglaLep1_0020	AQHT01039050.1	G/T	4145-4349	205	96/98
AglaLep1_0021	AQHT01028543.1	A/T	6794-7001	208	98/99
AglaLep1_0022	AQHT01085189.1	A/T	3526-3318	209	95/99
AglaLep1_0023	AQHT01083370.1	T/T	22936-22730	207	95/98
AglaLep1_0024	AQHT01078248.1	A/T	20909-20706	204	96/99
AglaLep1_0025	AQHT01071290.1	A/C	19810-19602	209	99/99
AglaLep1_0026	AQHT01061553.1	A/T	597-805	209	99/99
AglaLep1_0027	AQHT01060381.1	A/T	30190-29982	209	99/99
AglaLep1_0028	AQHT01058665.1	A/T	15976-15768	209	99/99
AglaLep1_0029	AQHT01057349.1	A/T	6486-6694	209	99/99
AglaLep1_0030	AQHT01045254.1	G/T	630-422	209	99/99
AglaLep1_0031	AQHT01041893.1	A/T	80788-80994	207	96/98
AglaLep1_0032	AQHT01013175.1	A/T	1569-1361	209	98/98
AglaLep1_0033	AQHT01015247.1	A/T	1311-1107	205	96/98
AglaLep1_0034	AQHT01074359.1	A/T	26576-26368	209	99/99
AglaLep1_0035	AQHT01068167.1	A/T	18535-18327	209	98/98
AglaLep1_0036	AQHT01055253.1	A/T	28392-28184	209	99/99

AglaLep1_0037	AQHT01053331.1	A/T	14613-14405	209	99/99
AglaLep1_0038	AQHT01033776.1	A/T	6379-6587	209	98/98
AglaLep1_0039	AQHT01032475.1	T/T	6836-6628	209	99/99
AglaLep1_0040	AQHT01028517.1	A/T	3020-3228	209	98/98
AglaLep1_0041	AQHT01017574.1	G/T	3856-4064	209	99/99
AglaLep1_0042	AQHT01008889.1	A/T	10437-10645	209	99/99
AglaLep1_0043	AQHT01005808.1	A/T	3847-3639	209	98/98
AglaLep1_0044	AQHT01036555.1	A/T	9119-8913	210	98/99
AglaLep1_0045	AQHT01074793.1	T/T	19845-19680	166	73/98
AglaLep1_0046	AQHT01066093.1	T/T	2402-2191	212	97/98
AglaLep1_0047	AQHT01042690.1	T/T	10855-11058	204	95/97
AglaLep1_0048	AQHT01041947.1	A/T	45321-45530	210	93/97
AglaLep1_0049	AQHT01085874.1	A/T	3779-3572	208	99/99
AglaLep1_0050	AQHT01043264.1	A/T	18013-17806	208	98/98
AglaLep1_0051	AQHT01036555.1	A/T	9723-9529	198	91/97
AglaLep1_0052	AQHT01036132.1	A/T	1102-890	213	97/99
AglaLep1_0053	AQHT01034806.1	A/T	477-684	208	99/99
AglaLep1_0054	AQHT01023912.1	A/T	10522-10734	213	97/99
AglaLep1_0055	AQHT01012340.1	T/T	997-779	219	95/99
AglaLep1_0056	AQHT01007401.1	A/T	9258-9051	208	98/99
AglaLep1_0057	AQHT01005829.1	A/T	12460-12666	208	98/99
AglaLep1_0058	AQHT01087541.1	G/T	28540-28333	208	97/98
AglaLep1_0059	AQHT01072655.1	A/T	5533-5325	209	98/98
AglaLep1_0060	AQHT01065399.1	A/T	6482-6274	209	98/98
AglaLep1_0061	AQHT01054183.1	T/T	10519-10311	209	98/98
AglaLep1_0062	AQHT01051520.1	A/T	292-500	209	98/98
AglaLep1_0063	AQHT01048575.1	A/T	888-1096	209	98/98
AglaLep1_0064	AQHT01043343.1	A/T	3249-3041	209	98/98
AglaLep1_0065	AQHT01031002.1	T/T	8169-7961	209	97/97
AglaLep1_0066	AQHT01026131.1	A/T	14806-15014	209	98/98
AglaLep1_0067	AQHT01014073.1	A/T	1827-2036	209	98/98
AglaLep1_0068	AQHT01011140.1	A/T	22684-22476	209	98/98
AglaLep1_0069	AQHT01008685.1	A/T	12541-12749	209	98/98
AglaLep1_0070	AQHT01076678.1	A/T	3484-3309	176	83/99
AglaLep1_0071	AQHT01006286.1	T/T	39485-39272	213	96/98
AglaLep1_0072	AQHT01006185.1	A/T	7580-7788	209	98/98
AglaLep1_0073	AQHT01080139.1	A/T	5864-6078	214	97/99
AglaLep1_0074	AQHT01053111.1	T/T	9008-8796	213	97/99
AglaLep1_0075	AQHT01035419.1	A/T	36141-36351	210	98/98
AglaLep1_0076	AQHT01034184.1	A/T	3710-3504	207	98/99
AglaLep1_0077	AQHT01018547.1	A/T	25373-25582	210	98/98
AglaLep1_0078	AQHT01000718.1	A/T	4307-4103	205	95/97
AglaLep1_0079	AQHT01085813.1	A/T	997-1205	209	97/97
AglaLep1_0080	AQHT01083962.1	T/T	1939-2147	209	98/98

AglaLep1_0081	AQHT01075570.1	T/T	11316-11109	208	98/99
AglaLep1_0082	AQHT01071865.1	A/T	691-483	209	98/98
AglaLep1_0083	AQHT01067620.1	A/T	601-809	209	98/98
AglaLep1_0084	AQHT01067574.1	G/T	6160-6368	209	97/97
AglaLep1_0085	AQHT01057498.1	A/T	12008-12216	209	98/98
AglaLep1_0086	AQHT01046975.1	A/T	11158-11366	209	98/98
AglaLep1_0087	AQHT01039329.1	C/T	13926-13716	210	97/97
AglaLep1_0088	AQHT01036444.1	T/T	12395-12605	209	98/98
AglaLep1_0089	AQHT01027913.1	T/T	20011-19803	209	96/97
AglaLep1_0090	AQHT01025035.1	G/T	6706-6914	209	98/98
AglaLep1_0091	AQHT01013662.1	T/T	1939-2147	209	98/98
AglaLep1_0092	AQHT01012702.1	T/T	5847-5639	209	98/98
AglaLep1_0093	AQHT01007667.1	A/T	3032-3240	209	97/97
AglaLep1_0094	AQHT01005303.1	A/T	12299-12091	209	98/98
AglaLep1_0095	AQHT01003613.1	T/T	32447-32239	209	97/97
AglaLep1_0096	AQHT01002928.1	A/T	5241-5449	209	97/97
AglaLep1_0097	AQHT01000064.1	A/T	2313-2521	209	97/97
AglaLep1_0098	AQHT01087238.1	A/T	4340-4229	212	97/98
AglaLep1_0099	AQHT01083296.1	T/T	3273-3477	207	98/99
AglaLep1_0100	AQHT01056026.1	A/T	30926-30717	210	98/98
AglaLep1_0101	AQHT01039455.1	A/T	4390-4600	210	98/98
AglaLep1_0102	AQHT01016488.1	T/T	2150-1939	212	95/98
AglaLep1_0103	AQHT01021711.1	A/T	4238-4037	202	94/98
AglaLep1_0104	AQHT01022525.1	A/T	12078-12229	262	78/98
AglaLep1_0105	AQHT01017379.1	A/T	15-225	211	97/98
AglaLep1_0106	AQHT01011585.1	T/T	21403-21196	202	97/98
AglaLep1_0107	AQHT01082695.1	A/T	4199-3991	209	97/97
AglaLep1_0108	AQHT01071943.1	T/T	35063-34855	209	97/97
AglaLep1_0109	AQHT01070124.1	A/T	4423-4212	212	97/99
AglaLep1_0110	AQHT01053188.1	A/T	2223-2431	209	97/97
AglaLep1_0111	AQHT01024499.1	A/T	14237-14029	209	97/97
AglaLep1_0112	AQHT01022938.1	T/T	421-629	209	97/97
AglaLep1_0113	AQHT01012770.1	A/T	758-546	213	95/97
AglaLep1_0114	AQHT01011713.1	A/T	4642-4844	203	95/98
AglaLep1_0115	AQHT01010850.1	A/T	4725-4933	209	96/96
AglaLep1_0116	AQHT01003477.1	A/T	10283-10075	209	97/97
AglaLep1_0117	AQHT01002817.1	A/T	25728-25936	209	97/97
AglaLep1_0118	AQHT01068396.1	A/T	1184-1391	208	97/97
AglaLep1_0119	AQHT01034412.1	A/T	76682-76868	186	86/97
AglaLep1_0120	AQHT01074302.1	A/T	8311-8504	194	92/99
AglaLep1_0121	AQHT01043758.1	A/T	12311-12103	209	97/97
AglaLep1_0122	AQHT01039808.1	G/T	9413-9625	213	94/96
AglaLep1_0123	AQHT01027387.1	A/T	28041-28229	188	84/95
AglaLep1_0124	AQHT01027515.1	A/T	5545-5340	206	97/99

AglaLep1_0125	AQHT01019184.1	A/T	260-466	207	97/98
AglaLep1_0126	AQHT01069318	A/T	1913-1717	197	93/99
AglaLep1_0127	AQHT01065775.1	G/T	5223-5016	208	97/97
AglaLep1_0128	AQHT01057921.1	A/T	14699-14901	203	94/97
AglaLep1_0129	AQHT01024600.1	A/T	16978-16770	209	96/96
AglaLep1_0130	AQHT01013319.1	A/T	655-862	208	97/97
AglaLep1_0131	AQHT01049250.1	A/T	2228-2444	217	95/99
AglaLep1_0132	AQHT01007250.1	T/T	2576-2776	201	94/97
AglaLep1_0133	AQHT01041900.1	T/T	319-112	208	97/97
AglaLep1_0134	AQHT01015448.1	A/T	26407-26201	207	96/97
AglaLep1_0135	AQHT01075514.1	A/T	8301-8093	209	95/95
AglaLep1_0136	AQHT01039118.1	A/T	4114-3928	187	86/97
AglaLep1_0137	AQHT01024038.1	A/T	2749-2957	209	96/96
AglaLep1_0138	AQHT01023919.1	A/T	774-980	207	93/96
AglaLep1_0139	AQHT01013379.1	A/T	328-530	203	95/98
AglaLep1_0140	AQHT01067492.1	A/T	8970-9182	213	95/97
AglaLep1_0141	AQHT01063623.1	A/T	17630-17421	210	95/97
AglaLep1_0142	AQHT01016233.1	A/T	630-841	212	97/99
AglaLep1_0143	AQHT01039823.1	A/T	3738-3946	209	95/95
AglaLep1_0144	AQHT01019436.1	A/T	16678-16492	187	89/100
AglaLep1_0145	AQHT01016654.1	A/T	2913-2721	193	92/99
AglaLep1_0146	AQHT01002699.1	A/T	4341-4132	210	95/99
AglaLep1_0147	AQHT01083304.1	A/T	25350-25554	205	96/99
AglaLep1_0148	AQHT01023720.1	A/T	8491-8295	197	93/98
AglaLep1_0149	AQHT01017158.1	G/T	2282-2075	208	96/96
AglaLep1_0150	AQHT01014775.1	A/T	16675-16470	206	95/96
AglaLep1_0151	AQHT01083612.1	A/T	2712-2919	208	96/96
AglaLep1_0152	AQHT01007081.1	T/T	3991-4175	185	86/97
AglaLep1_0153	AQHT01059643.1	A/T	2961-2759	203	94/97
AglaLep1_0154	AQHT01059917.1	A/T	48141-48353	208	95/97
AglaLep1_0155	AQHT01080651.1	A/T	3700-3504	197	90/96
AglaLep1_0156	AQHT01087984.1	A/T	1599-1801	203	93/95
AglaLep1_0157	AQHT01080093.1	A/T	196-395	200	95/99
AglaLep1_0158	AQHT01058523.1	A/T	9818-9619	200	95/99
AglaLep1_0159	AQHT01040918.1	A/T	2383-2181	203	94/97
AglaLep1_0160	AQHT01038470.1	A/T	14356-14560	205	94/96
AglaLep1_0161	AQHT01018394.1	T/T	1256-1050	207	91/96
AglaLep1_0162	AQHT01058645.1	A/T	6406-6619	214	88/96
AglaLep1_0163	AQHT01007467.1	A/T	12989-13205	217	94/97
AglaLep1_0164	AQHT01086529.1	A/T	7936-7738	199	91/96
AglaLep1_0165	AQHT01021667.1	A/T	2220-2476	197	93/98
AglaLep1_0166	AQHT01062527.1	A/T	50644-50447	198	92/97
AglaLep1_0167	AQHT01015142.1	A/T	2009-1807	203	94/97
AglaLep1_0168	AQHT01024974.1	A/T	4708-4509	200	92/96

AglaLep1_0169	AQHT01064561.1	T/T	30313-30508	196	92/98
AglaLep1_0170	AQHT01090934.1	A/T	14287-14528	241	84/98
AglaLep1_0171	AQHT01014494.1	A/T	490-690	201	94/98
AglaLep1_0172	AQHT01005868.1	A/T	74-285	212	90/97
AglaLep1_0173	AQHT01079708.1	T/T	19936-19733	204	94/97
AglaLep1_0174	AQHT01054319.1	C/T	9182-9394	213	93/95
AglaLep1_0175	AQHT01057097.1	A/T	4863-4669	195	93/99

Table S5 Blastx analysis of the flanking sequences of the representative non-lepidopteran *Lep*1 elements.

Name	5'-Flanking sequence			3'-Flanking sequence		
	Description (Gene function/ organism)	GenBank Accession No.	E-value	Description (Gene function/ organism)	GenBank Accession No.	E-value
CvBVLep1_1	hypothetical protein BV9-4/ Cotesia congregata bracovirus	CCB96410.1	1e-61	SER-RICH protein/ Cotesia sesamiae Kitale bracovirus (9265 bp available)	CCQ19230.1	1e-61
CvBVLep1_2	protein tyrosine phosphatase/ Cotesia plutellae polydnavirus (6082 bp available)	AAZ04267.1	0.0	PTP-kappa/ Cotesia sesamiae Kitale bracovirus (7437 bp available)	CCQ19288.1	0.0
CvBVLep1_3	hypothetical protein/ Cotesia plutellae polydnavirus (7650 bp available)	ABK63311.1	1e-15	conserved hypothetical protein BV8/ Cotesia sesamiae Kitale bracovirus	CCQ19261.1	6e-58
CsKBVLep1_1	putative 5'-nucleotidase NT5-like2/ Cotesia congregata bracovirus	CCQ71110.1	5e-166	putative 5'-nucleotidase NT5-like4/ Cotesia congregata bracovirus	CCQ71112.1	0.0
CsKBVLep1_2	conserved hypothetical protein/ Cotesia vestalis bracovirus	AEE09583.1	0.0	conserved hypothetical protein/ Cotesia vestalis bracovirus	AEE09551.1	8e-73
CsKBVLep1_3	conserved hypothetical protein/ Cotesia vestalis bracovirus	AEE09576.1	3e-48	EP1-like protein/Cotesia vestalis bracovirus	AEE09456.1	1e-124
CsKBVLep1_4	conserved hypothetical protein/ Cotesia vestalis bracovirus	AEE09571.1	1e-152	hypothetical protein BV6-18/ Cotesia congregata bracovirus (3724 bp available)	CCQ71245.1	8e-45
CsMBVLep1_1	hypothetical protein BV9-4/ Cotesia congregata bracovirus (5058 bp available)	CCB96410.1	2e-60	conserved hypothetical protein/ Cotesia vestalis bracovirus	AEE09551.1	4e-67
CsMBVLep1_2	conserved hypothetical protein/ Cotesia vestalis bracovirus	AEE09575.1	2e-47	PREDICTED: hypothetical protein LOC100570266/ Acyrthosiphon pisum	XP_003245995.1	0.0
CsMBVLep1_3	conserved hypothetical protein/ Cotesia vestalis bracovirus	AEE09480.1	4e-147	hypothetical protein/ Cotesia plutellae polydnavirus	ABK63349.1	2e-149
CsMBVLep1_4	viral ankyrin/ Cotesia vestalis bracovirus (4703 bp available)	AEE09523.1	2e-90	protein tyrosine phosphatase/ Cotesia vestalis bracovirus	AEE09520.1	0.0
AP_Lep1_1	hypothetical protein LOC100574170/ Acyrthosiphon pisum (only4990 bp available)	XP_003241665.1	0.0	hypothetical protein LOC100574170/ Acyrthosiphon pisum (only 2045 bp available)	XP_003241665.1	2e-34
AP_Lep1_2	PREDICTED: suppressor of tumorigenicity 14 protein-like/ Bombus impatiens	XP_003491086.1	1e-10	hypothetical protein YQE_01804, partial/ Dendroctonus ponderosae	ENN81797.1	4e-11
AP_Lep1_3	No significant hit			No significant hit		
AP_Lep1_4	hypothetical protein TcasGA2_TC006067/ Tribolium castaneum	EFA08420.1	4e-44	PREDICTED: zinc finger, MYM domain containing 1/ Oryctolagus cuniculus	XP_002720742.1	2e-11
AP_Lep1_5	homeobox protein koza-like/ Acyrthosiphon pisum	XP_003242054.1	7e-4	PREDICTED: similar to CG11085 CG11085-PA/ Tribolium castaneum	XP_972824.1	4e-23
AglaLep1_0001	No significant hit (only 956 bp available)			No significant hit (only 1687 bp available)		
AglaLep1_0002	PREDICTED: zinc finger MYM-type protein 1-like/ Hydra vulgaris (8152 bp available)	XP_004207220.1	8e-09	PREDICTED: hypothetical protein LOC100740015/ Bombus impatiens	XP_003488412.1	3e-65
AglaLep1_0003	Transposase/ Tapinoma nigerrimum	CCC86609.1	4e-42	hypothetical protein TcasGA2_TC006450/ Tribolium castaneum	EFA08763.1	1e-147
AglaLep1_0004	hypothetical protein X777_00501/ Cerapachys biroi	EZA59380.1	9e-43	No significant hit (only 533 bp available)		
AglaLep1_0005	hypothetical protein X777_11794/ Cerapachys biroi	EZA49708.1	2e-36	PREDICTED: similar to Y26D4A.11/ Tribolium castaneum	XP_001810658.1	9e-21

Note: Only flanking sequences extracted from the BAC clone sequences were subject to analysis. Other flanking sequences of *CvLep*1, *NbLep*1, *CfLep*1 and *AgosLep*1 are generally short and are not included in analysis.

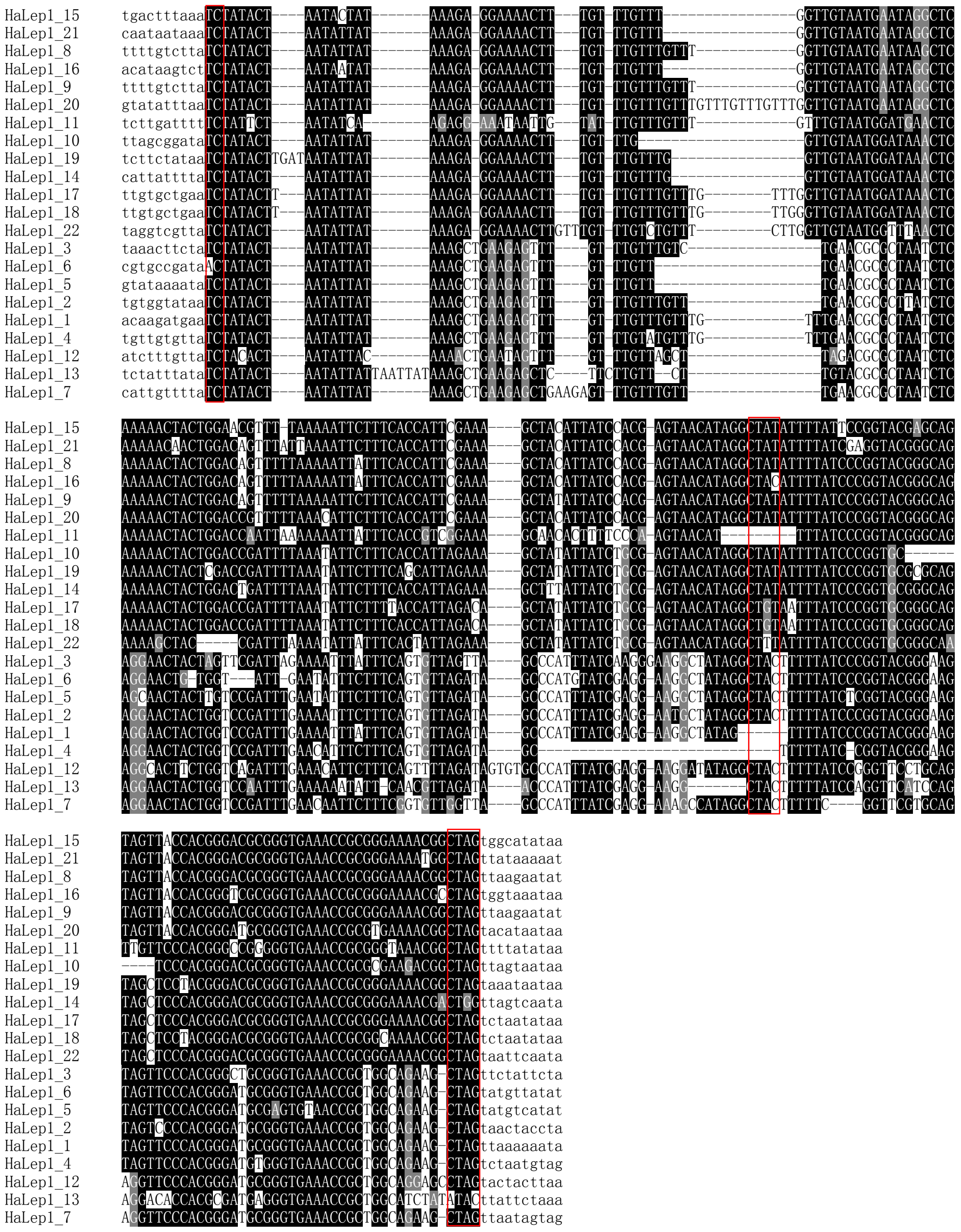

Figure S1

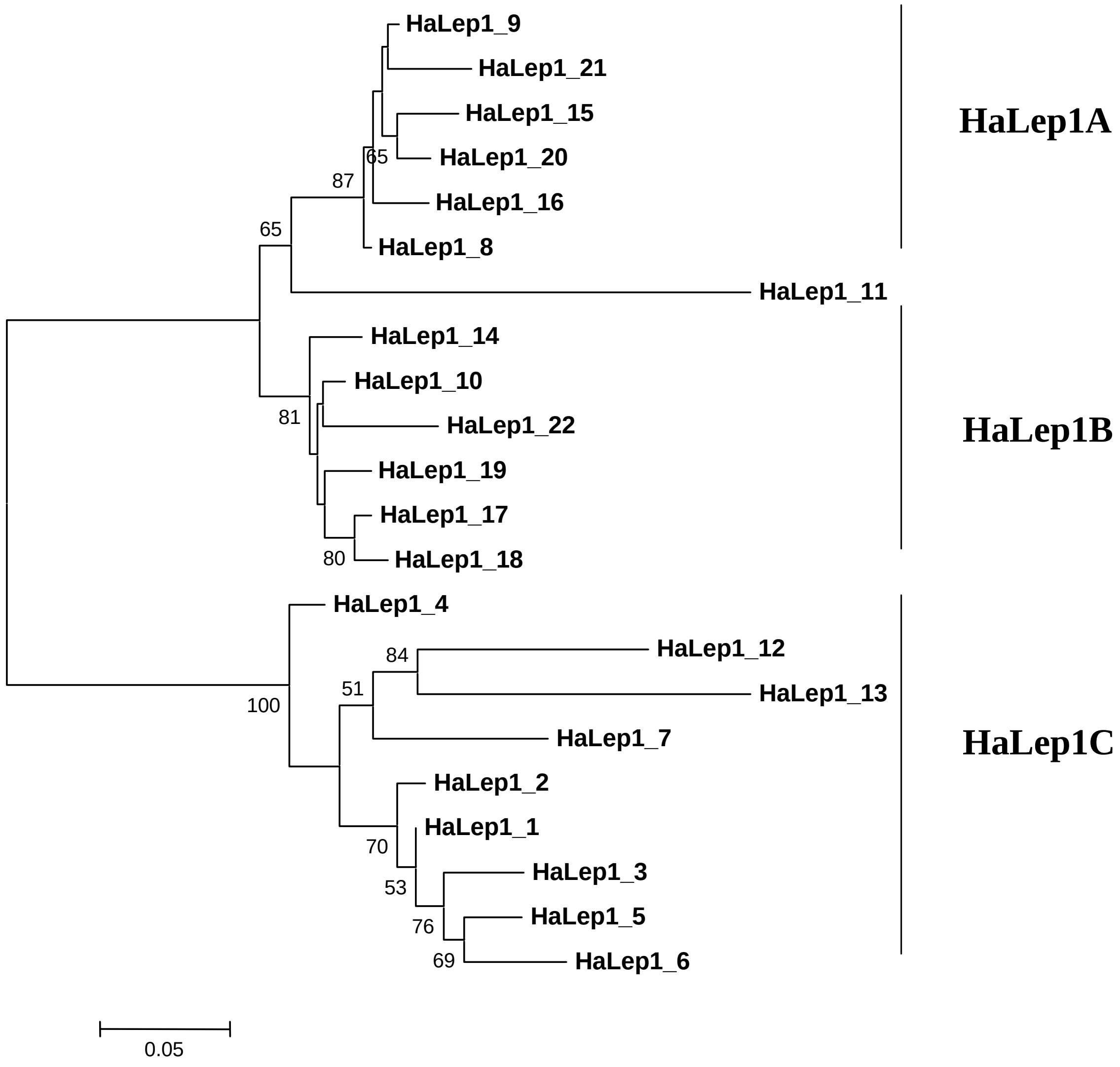
HaLep1_9
HaLep1_21
HaLep1_15
HaLep1_20
HaLep1_16
HaLep1_8
HaLep1_11
HaLep1_14
HaLep1_10
HaLep1_22
HaLep1_19
HaLep1_17
HaLep1_18
HaLep1_4
HaLep1_12
HaLep1_13
HaLep1_7
HaLep1_2
HaLep1_1
HaLep1_3
HaLep1_5
HaLep1_6
65
87
65
81
80
84
51
100
70
53
76
69
HaLep1A
HaLep1B
HaLep1C
0.05

Figure S2

```
HaLep1_1    acaagatgaaTCTATAC----TAATATTATAAAGCTGAAGAGTTTGTTTGTTTGTTTGTTTGAACGCGCTAATCTCAGGAACTACTGGTCCGATTTGAAA
EF152213    tttatcctgaTCCATAC----TAATATTATAAAGCTGAAGAGTTTGTTTGTTTGTTTG----AATGCGCTAATCTCAGGAACTACTGGTCCGATTTGAAA
EF152207    agttttggcaTCTATAC----TAATATTATAAAGCTGAAGAGTTTGTTTGTTTGTTT----GAACGCGCTAATCTCAGGAACTACTGGTCCGATTTGAAC
HQ840515    tttatacttaTCTATAC----TAATATTATAAAGCTGAAGAGTTTGTTTGTTTGTTTATTTGAACGCGCTAATCTCAGGAACTACTGGTCCGATTTGAAC
GT133743    tagcatattaTCTATAC----TAATATTATAAAGCTGAAGAGTTTGTTTGTGTGTTTGTTTGAACGCCCTAATCTCAGGAACTACTGGTCCGGTTTGAAA
GR964554    aagtaacctaTCTATACCTACTAATATTATAAAGCTGAAGAGTTTGTTTGT----TTGTTTGAACGCGCTAATCTCAGGAAGTACTGGTCCGATTTGAAA
GT133396    gttatacttaTCTATAC----TAATATTATAAAGCTGAAGAGTTTGTTTGT----TTG----AACGCGCTAATCTCAGGAACTACTGGTCCGATTTGAAA

HaLep1_1    ATTTATTTCAGTGTTAGATAGCCCATTTATCGAGGAAGGCTATAG------TTTTATCCCGGTACGGGAAGTAGTTCCCACGGGAT-GCGGGTGAAACCG
EF152213    ATTTCTTTCAGTGTTAGATAGCCCATTTATCGAGGAAGGCTATAGGTTACTTTTTATCCCGGTACGGGAAGTAGTTTCCACGGGAT-GCGGGTGCAACCG
EF152207    AATTCTTTCGGTGGTAGATAGCCCATTTATCGAGGAAGGCTATAGGCTACTTTTTATCCAGGTTCGTGCAGAGGTTCCCACGGGAT-GCGGGTGAAACCG
HQ840515    AATTCTTTCGGTGTTAGATAGCCCATTTATC---------------------------CGGGTTCGTGCAGAGGTTCCCACGTGAT-GCGGGTGAAACCG
GT133743    AATGATTTCAGTGTTAGATAGCCCATTTATCGAGGAAGGCTATAGGCTACTTTTTATCCGGGTGCGCCGAGTAGTTCCCACGGGAAAGCGGGTGAAACCA
GR964554    AATTCTTTCAGTGTTAGATAGCCCATTTATCGAGGAAGGCTATAGGCTACTTTTTATCCGGGTGCGCGCAGTAGTTCCCACGGGATAGCGGGTGAAACCG
GT133396    AATTCTTTCAGTGTTAGATAGCCCATTTATCGAGGAAGGCTATAGGCT---TTTTATCCGGGTGCGCGCAGTTGTTCCCACGGGAAAGCGGGTGAAACCG

HaLep1_1    CTGGCAGAAGCTAGttaaaaaata
EF152213    CTGGCAGAAGCT--
EF152207    CTGGCAGAAGCTA-
HQ840515    CTGGCAGAAGCTAGtaatcattaa
GT133743    CTGGCGGAAGCTAGtacaatatat
GR964554    CTGGCGGAAGCTAGttgattcatg
GT133396    TTGGCAGAAGCTAGtttaaaataa
```

Figure S3

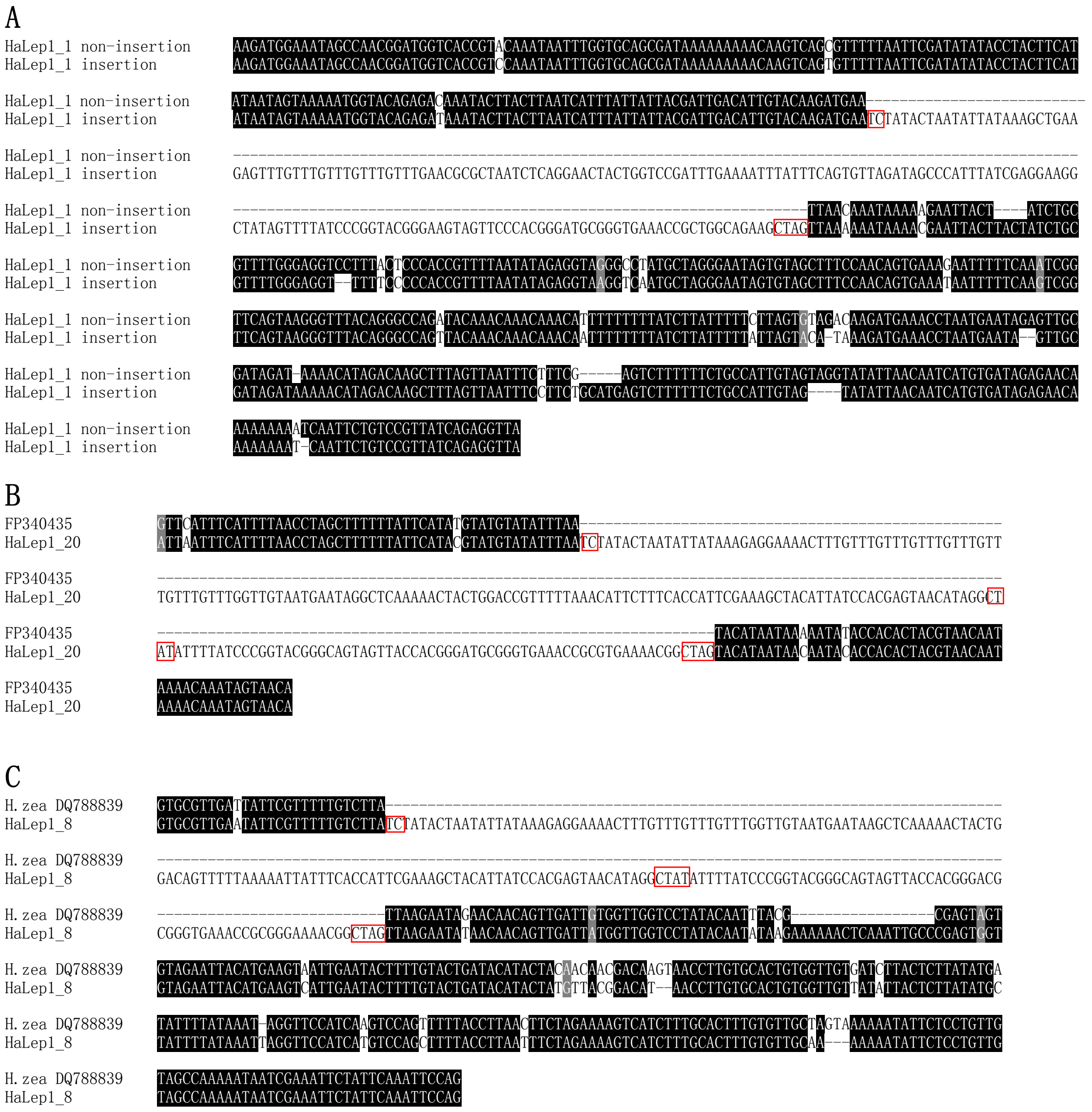
A
HaLep1_1 non-insertion
HaLep1_1 insertion
B
FP340435
HaLep1_20
C
H.zea DQ788839
HaLep1_8

Figure S4

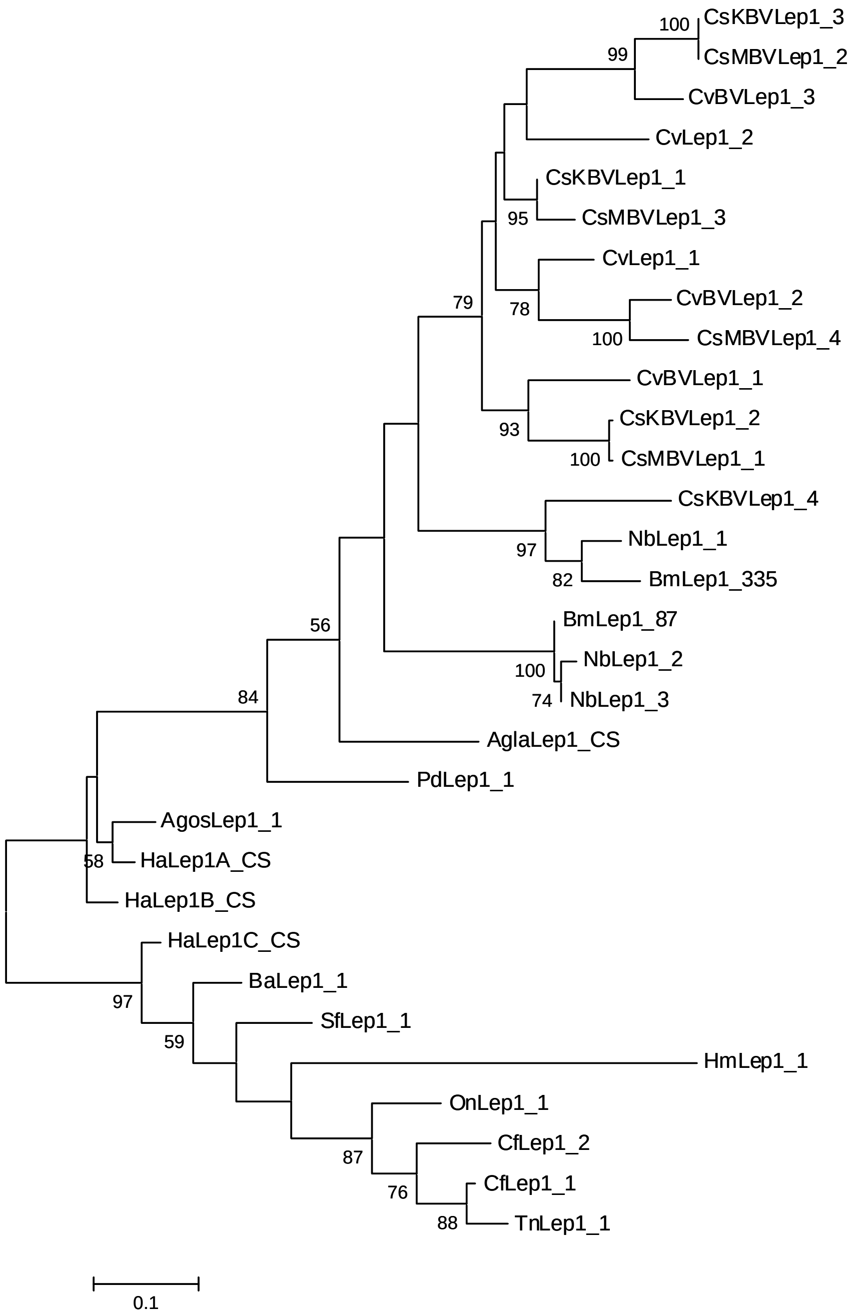
CsKBVLep1_3
CsMBVLep1_2
CvBVLep1_3
CvLep1_2
CsKBVLep1_1
CsMBVLep1_3
CvLep1_1
CvBVLep1_2
CsMBVLep1_4
CvBVLep1_1
CsKBVLep1_2
CsMBVLep1_1
CsKBVLep1_4
NbLep1_1
BmLep1_335
BmLep1_87
NbLep1_2
NbLep1_3
AglaLep1_CS
PdLep1_1
AgosLep1_1
HaLep1A_CS
HaLep1B_CS
HaLep1C_CS
BaLep1_1
SfLep1_1
HmLep1_1
OnLep1_1
CfLep1_2
CfLep1_1
TnLep1_1
100
99
95
79
78
100
93
100
97
82
56
100
74
84
58
97
59
87
76
88
0.1

Figure S5

```
CsKBV_Lep1_4      ttacgtgataTCTATACAAATATTATAAAGAGGAAAGGTTTGATTTTTTGTTTGTTTGTTTGTTTGTATGAATTGAATAGGCTCCGAAACTACTTGGCAG
BmLep1_335        atagaatataTCTATACTAATATTATAAAGAGGAAAGATTT------------GTTTGTTTGTTTGTAT----TGAATAAGCTCCGAAACTACTGAACCG

CsKBV_Lep1_4      ATTTGAAAAATTCTTTCACTGTTGGAAAGCTACATCATTCCTGAGTGACATAGGCTATATTTCATTTTCAAAAAAAA-TAGGCATCCTTACTAAAATTAC
BmLep1_335        ATTTGAAAAATTCCTTCACTGTTTGGAAGCTACATCATTCGCGAGTGACTTAGGCTATAATCTTTTTTGAAAAAAAAATAGTGCTCTTTACTAAAACTCC

CsKBV_Lep1_4      GATAACGTAATCCAAGGTGTAAAAAAAATATCAAAAAACTATC-TTACATCGCGTGCGCTGCGAAAACTATTGATGATAGAACAAAATGATATACTACAT
BmLep1_335        AGTAATGTAACCCAAGGTGTAAAAAAAATTA-CATAAAATATTCATTACACCGGGTGCCCTGCGAAAACTATTGATGATAGAACAAAATAATGTACTACAA

CsKBV_Lep1_4      TTTTTTAGAACACACCATTATCTACAAAAAAT-CAGCGGCAGCATATCTCTAACTATtacagtcatg-----------------------------------
BmLep1_335        CTTTGTAGAACACATTATTATTTACAAAAAGTGTCGCGACAGCATATGTCTAACTATTATAGTTATGCCGCAATAAGAGTTCTTTTATTTTTTTAAATAA

CsKBV_Lep1_4       -------------------------------------------------------------------------
BmLep1_335        AACAACGTCAAATATCGTTGAAATTTTTGTTAAAGACCCGAGCGGAGCCGGAGCGGACCGCTAGtagacaataa
```

Figure S6
